# Spatio-Temporal and Cultivar-Dependent Variations in the Cannabis Microbiome

**DOI:** 10.3389/fmicb.2020.00491

**Published:** 2020-03-24

**Authors:** Dominique Comeau, Amy Novinscak, David L. Joly, Martin Filion

**Affiliations:** Department of Biology, University of Moncton, Moncton, NB, Canada

**Keywords:** Cannabis, cannabinoids, microbiome, rhizosphere, endorhizosphere, phyllosphere

## Abstract

The incipient legalization and commercialization of *Cannabis sativa* in Canada have promulgated research into characterizing the plant’s microbiome as it promotes many facets of plant growth and health. The emblematic production of commercially important secondary metabolites, namely tetrahydrocannabinol (THC), cannabidiol (CBD) and terpenes, has warranted investigating the modulating capacity of these molecules on the plant microbiome. *C. sativa* cultivars can be classified into chemotypes depending on the relative levels of THC and CBD they produce; their biosynthesis also varies spatially and temporally during the life cycle of the plant. To study the differential microbiome structure and diversity between cultivars in a spatio-temporal manner, we extracted microbial DNA from the rhizosphere, endorhizosphere, and phyllosphere during the entire life cycle of three different chemotypes; CBD Yummy (<1% THC/13% CBD), CBD shark (6% THC/10% CBD) and Hash (14% THC/ < 1% CBD). Illumina marker gene sequencing of bacterial (16S) and fungal (ITS) communities were coupled to the QIIME2, PICRUSt, and LEfSe pipelines for analysis. Our study describes spatio-temporal and cultivar-dependent variations in the fungal and bacterial microbiome of *C. sativa*, and details strong cultivar-dependent variance in the belowground microbiome. Furthermore, the predicted pathway abundance of the bacterial microbiome is concomitantly subject to spatio-temporal variations; pathways related to lipid, amino acid, glucose and pentose metabolism were noteworthy. These results describe, for the first time, spatio-temporal and cultivar-dependent variations in the microbiome of *C. sativa* produced under strict commercial settings. Describing the microbiome is the first step in discoveries that could help in engineering a plant growth and health promoting microbiome in future works.

## Introduction

*Cannabis sativa* is increasingly being produced under commercial conditions largely due to its decriminalization in Uruguay, several U.S. States and more recently in Canada (Small, 2017). *C. sativa* is mainly known for its glandular trichomes that adorn the aerial epidermis and produce various cannabinoids, terpenes, and phenolics (Kim and Mahlberg, 1997; Elsohly and Slade, 2005; Flores-Sanchez et al., 2012; Staginnus et al., 2014). The allogamous nature of the plant has left its taxonomical classification a contentious issue; nevertheless, the plant has been successfully separated into chemical phenotypes (chemotypes) based on the differential levels of cannabinoids produced, namely tetrahydrocannabinol (THC) and cannabidiol (CBD) (Hillig and Mahlberg, 2004; Piomelli and Russo, 2016). Although the classification does not yet take into account other secondary metabolites contributing to the chemical profiles, they have been shown to vary accordingly (Aizpurua-Olaizola et al., 2016). The 565 identified constituents of the trichome resin are comprised of 120 phyto-cannabinoids and their derivatives, many of which have been shown to fluctuate temporally during the life cycle of the plant (Aizpurua-Olaizola et al., 2016; ElSohly et al., 2017). Spatially, the cannabinoids are highly concentrated on the inflorescence at plant maturity and have a nominal presence on the roots (Slatkin et al., 1971; Small, 2017; Gul et al., 2018). Due to their cytotoxicity, the cannabinoids are synthesized extracellularly in the trichome lumen and accumulate in the resin (Sirikantaramas et al., 2005). The cannabinoids may act as repellents or as chemotaxis molecules (Sirikantaramas et al., 2004, 2007; Morimoto et al., 2007). In fact, one of the many ways in which plants advantageously modulate their microbiome is through the secretion of secondary metabolites that attract and repel distinct microorganisms, not dissimilar to the secondary metabolites produced in the resin of the trichomes (Bais et al., 2003; Walker et al., 2003; Oldroyd, 2013; Baetz and Martinoia, 2014; Huang et al., 2018). Hence, the secondary metabolites produced by *C. sativa* could demonstrably alter the plant microbiome in a chemotype-dependent manner as well as spatially and temporally.

The total plant microbiome can be sectioned into distinct microenvironments having distinct microbiomes that also fluctuate temporally along with the life cycle of the plant (van Overbeek and van Elsas, 2008; Lundberg et al., 2012; Chaparro et al., 2014). In other words, the microbiome of each microenvironment is subject to different biotic and abiotic cues and stressors that dynamically shape the microbiome structure and function (Reinhold-Hurek and Hurek, 2011; Bulgarelli et al., 2012; Lundberg et al., 2012; Vokou et al., 2012; Vorholt, 2012; Bodenhausen et al., 2013; Chaparro et al., 2013; Muller and Ruppel, 2014; Edwards et al., 2015; Zhalnina et al., 2018). These microenvironments include, but are not limited to, the rhizosphere (immediate area of soil surrounding the roots), the phyllosphere (aerial plant surfaces) and the endorhizosphere (within plant roots) (Bulgarelli et al., 2013). The endorhizosphere microbiome of *C. sativa* was shown to fluctuate between chemotypes but maintained a core microbiome composed of the genus *Pseudomonas*, *Cellvibrio*, *Oxalobacteraceae*, *Xanthomonadaceae*, *Actinomycetales*, and *Sphingobacteriales* (Winston et al., 2014). Another study noted a high fungal diversity on the inflorescence of high THC chemotypes and identified *Penicillium copticola*, belonging to the class Ascomycota, as the dominant fungal species of the phyllosphere (Kusari et al., 2012). Conversely, in two low-THC/high-CBD chemotypes, the phyllosphere was shown to be primarily populated by the *Pseudomonas*, *Panteo*, and *Bacillus* bacterial genera, as well as the *Aureobasidium*, *Alternaria*, and *Cochliobolus* fungal genera (Scott et al., 2018). Intriguingly, although cannabinoids have not been detected in high abundance in the root system, they do exude appreciable amounts of terpenes and phenolic constituents that could potentially alter the microbiome structure (Slatkin et al., 1971; Sakakibara et al., 1995; Fischedick et al., 2010; Lesma et al., 2014).

To the best of our knowledge, spatio-temporal variations in the microbiome structure of *C. sativa* over the entire plant growth period have yet to be characterized. We speculated that a comprehensive study, executed under real commercial settings and using different cultivars varying in cannabinoids content could yield useful insight into the dynamic interplay between chemotypes and their microbiome structure. To characterize spatio-temporal variations in the microbiomes of three *C. sativa* chemotypes ranging from low to high THC and CBD content [CBD Yummy (<1% THC/13% CBD), CBD shark (6% THC/10% CBD) and Hash (14% THC/<1% CBD)], we utilized Illumina culture-independent marker gene sequencing of bacterial (16S) and fungal (ITS) communities coupled to the QIIME2, PICRUSt and LEfSe pipelines (Segata et al., 2011; Langille et al., 2013; Bolyen et al., 2019). The rhizosphere, endorhizosphere and phyllosphere were sampled during the plants’ propagation, pre-vegetative, early-flowering and late-flowering stages to highlight spatio-temporal variations during the entire growth period of the plant. We determined that these cultivars, which have distinct chemotypes, differently recruit their microbiomes in a spatio-temporal manner when produced under the same regulated commercial settings.

## Materials and Methods

### Chemotypes and Sampling

Three *C. sativa* chemotypes; CBD Yummy (less than 1% THC/13% CBD), CBD shark (6% THC/10% CBD) and Hash (14% THC/less than 1% CBD) grown in standard commercial production conditions were sampled from Organigram’s facility (Moncton, NB, Canada). The main terpene content of CBD Yummy includes pinene, caryophyllene, and myrcene. The terpene content of CBD Shark includes pinene, myrcene, limonene, linalool, and caryophyllene. As for the Hash, it contains terpenes include myrcene, caryophyllene, humulene, and limonene. The plants were cloned from single model plants by vegetative propagation. Using clones rather than seeds helps to circumvent genetic variations due to the plant’s promiscuous nature, which could incidentally have an impact on the microbiome (Chandra et al., 2017b).

The experimental set-up consisted of the three *C. sativa* chemotypes listed above and four harvesting periods corresponding to various growth stages. Samples were harvested from 10 randomly selected healthy plants for every chemotype at each growth stage. More specifically, the rhizosphere, phyllosphere, and endorhizosphere were sampled during the plants’ propagation, pre-vegetative, early-flowering and late-flowering stages (Supplementary Figure S1) (Chandra et al., 2017a). DNA was immediately extracted on-site after harvesting, quantified and sent for sequencing (Illumina platform). Since the plants at the propagation stage were not grown in a soil-based substrate, but rather in rock wool, we were unable to harvest appreciable amounts of DNA from the rhizosphere at this growth stage. However, the plants were subsequently transplanted in coconut based medium (Canna Coco; Toronto, ON, Canada) at the later growth stages making the rhizosphere amenable to DNA extraction. The root system of *C. sativa* at the later growth stages were highly dense and evenly distributed in the pots because of the orderly watering of the plant, which enabled us to use a sample core for rhizosphere extraction. Furthermore, because the microbiome is known to fluctuate along the length of the root system, the sample core enabled the extraction of superficial as well as deeper soil samples along the root architecture, assuring proper sampling of the entire rhizosphere microbiome (Folman et al., 2001). The rhizosphere samples consisted of soil adhering to the roots. The soil was then removed from the roots before the sampling of the endorhizosphere. To ensure sufficient coverage of the phyllosphere, each sample consisted of an amalgam of five individual leaves spanning the entire length of the plant at regular intervals. During the late-flowering stage, the inflorescence and sweet leaves which present a higher concentration of cannabinoids were sampled separately from leaf samples. This was done to better identify spatial variations in the microbiome of the phyllosphere dependent on cannabinoid concentrations. Although we were able to amplify fungal DNA from the phyllosphere (leaves, sweet leaves, and inflorescence), no appreciable amount of bacterial DNA could be amplified and therefore could not be included in the analysis.

The number of *C. sativa* plant samples analyzed per growth stages was: 20 for the propagation stage [endorhizosphere (*n* = 10) and leaves (*n* = 10)]; 30 for the pre-vegetative stage [rhizosphere (*n* = 10), endorhizosphere (*n* = 10) and leaves (*n* = 10)]; 30 for the early flowering stage [rhizosphere (*n* = 10), endorhizosphere (*n* = 10) and leaves (*n* = 10)], and finally 50 for the late flowering stage (rhizosphere (*n* = 10), endorhizosphere (*n* = 10), leaves (*n* = 10), sweet leaves (*n* = 10) and inflorescence (*n* = 10). In total130 samples were analyzed per cultivar and this was repeated for each of the 3 cultivars, namely CBD Yummy, CBD Shark and Hash for a grand total of 390 samples. DNA extractions were performed on these 390 samples, which were submitted to 16S and ITS amplification and sequencing. However, as only ITS could be amplified from the leaves, sweet leaves and inflorescences, 16S analyses were restricted to the belowground plant parts (rhizosphere and endorhizosphere) (Supplementary Figure S1).

### DNA Extraction and Metagenome Profiling

In all cases, fungal and bacterial DNA was extracted using the Qiagen DNA DNeasy plant mini extraction kit (Qiagen, Mississauga, Canada). The samples were first frozen in liquid nitrogen and disrupted in a Tissuelyser (Qiagen) at maximum speed before utilizing the DNA extraction kit. The quantity and quality of the isolated DNA was assessed with a Qubit fluorometer (Thermo Fisher, Mississauga, Canada). Subsequently, PCR amplification of the bacterial 16S rRNA (16S) and the fungal internal transcribed spacer (ITS) as well as the Illumina sequencing was performed by the McGill University and Genome Québec Innovation Centre. The 16S V4 region was amplified using the primer pair 515F/806R and the ITS region was amplified using the primer pair ITS1F/ITS2 (Gardes and Bruns, 1993; Caporaso et al., 2011). The raw paired-end reads from the McGill University and Genome Québec Innovation Centre were processed with the QIIME2 (version 2019.7) pipeline (Bolyen et al., 2019). DADA2 was used to assess the quality of the reads which included filtering, trimming, denoising, dereplicating, merging of the forward, and reverse strands as well as removing chimeras (Callahan et al., 2016). We obtained a total of 12,926,478 paired-end reads with 3097 features identified after quality filtering of ITS data and a total of 14,456,801 paired-end reads with 14,517 features after quality filtering of 16S data. Amplicon sequence variants (ASV) were aligned using mafft-plugin which was subsequently used for the fassttree2-plugin which was needed for the diversity analysis (Katoh et al., 2002; Price et al., 2010). Samples used in diversity metrics were rarefied to an appropriate sampling depth for analysis. The ITS and 16S rarefaction curves can be found in Supplementary Figures S2A,B, respectively. The average sequencing depth of the 16S and ITS data was 59852 and 54603, respectively. Alpha-diversity and statistics were calculated with the Shannon distance metric. Beta-diversity and statistics were calculated using the Bray–Curtis dissimilarity or the Jaccard similarity indexes and plotted using the Vega Editor (QIIME2). Taxonomy was assigned to the 16S data using a Naïve Bayes pre-trained Silva 132 99% OTU classifier bounded by the 515F/806R primer set (Quast et al., 2013). Taxonomy was assigned to the ITS by training a Naïve Bayes classifier from the UNITE sh_qiime_ver7_99_10.10.2017 reference reads and taxonomy which were bounded by the ITS1F/ITS2 primer set (Nilsson et al., 2019). Differences in the abundance of bacteria and fungi were calculated using linear discriminant analysis (LDA) effect size (LEFSe) and the predicted metagenome functions were calculated using the PICRUSt2 QIIME2 plugin (Segata et al., 2011; Langille et al., 2013). The heatmaps were generated by Plotly Technologies Inc.

All sequences generated in this study have been deposited in DDBJ/EMBL/GenBank under the BioSample Accessions: PRJNA595913.

### Statistical Analysis

All statistical tests were performed using the QIIME2 interface (Bolyen et al., 2019). More specifically, pairwise Kruskal–Wallis test was used for assessing statistical significance of alpha diversity (Shannon’s index) between several groups. Bray–Curtis distance metrics were subjected to permutational multivariant analysis of variance (PERMANOVA) to assess statistical significance of diversity between several group with a permutation number of 999. ANCOM was used to assess statistical differences in taxonomy and pathway abundance between groups (Mandal et al., 2015).

## Results

### Diversity Metrics and Taxonomy of the Cannabis Fungal Microbiome

**FIGURE 1:**
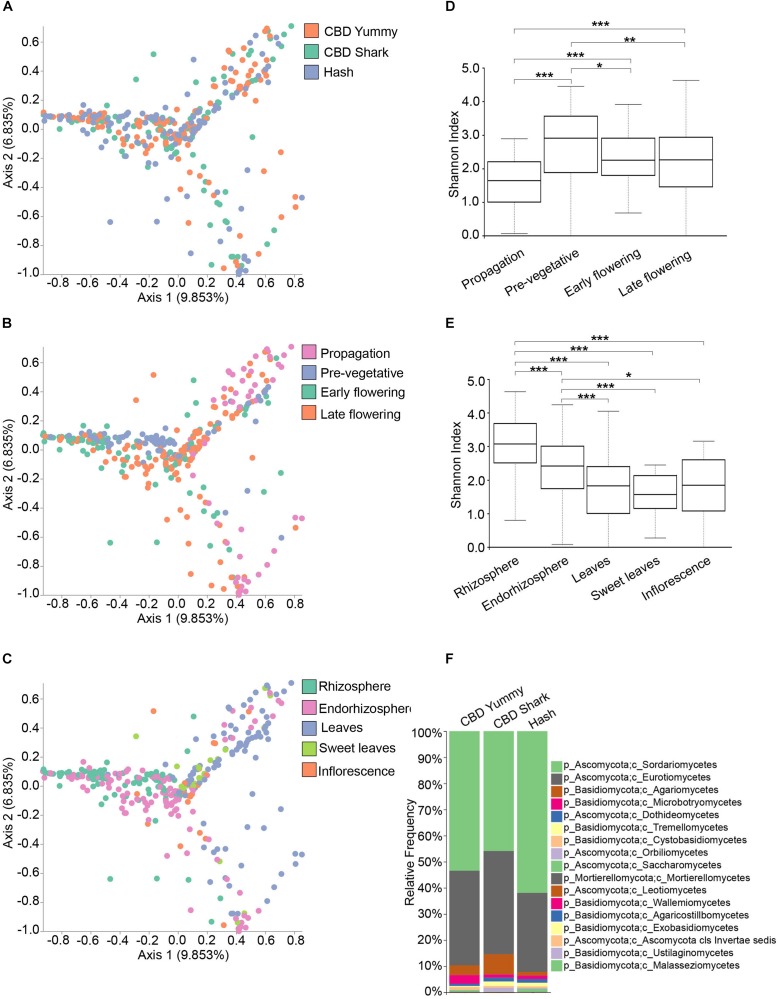
Diversity metrics and taxonomical differences in the fungal microbiome of cannabis. **(A)**. PCoA plot representative of inter-cultivar beta-diversity utilizing Bray–Curtis dissimilarity among samples (PERMANOVA: *R*^2^ = 0.013, *p* = 0.001). Each point represents a sample (CBD Yummy, *n* = 130; CBD Shark, *n* = 130; Hash, *n* = 130). **(B)**. PCoA plot representative of temporal beta-diversity utilizing Bray–Curtis dissimilarity among samples (PERMANOVA: *R*^2^ = 0.080, *p* = 0.001). Each point represents a sample (Propagation, *n* = 60; Pre-vegetative, *n* = 90; Early flowering, *n* = 90; Late flowering, *n* = 150). **(C)**. PCoA plot representative of spatial beta-diversity utilizing Bray–Curtis dissimilarity among samples (PERMANOVA: *R*^2^ = 0.091, *p* = 0.001). Each point represents a sample (Rhizosphere, *n* = 90; Endorhizosphere, *n* = 120; Leaves, *n* = 120; Sweet leaves, *n* = 30; Inflorescence, *n* = 30). For all PcoA plots, axis 1 and axis 2 represent the percentage of variance explained by each coordinate. **(D**,**E)** Alpha-diversity metric (Shannon’s index) of temporal and spatial variations amongst groups, respectively. Kruskal–Wallis pair-wise test was used to assess statistical significance between groups (***p < 10e-04,**p < 0.001,*p < 0.05; only statistically significant values are shown, non-significant groups are not represented). **(F)** Bar plot of the relative frequency of fungal taxa at the phylum and class level in the three chemotypes studied (CBD Yummy, *n* = 130; CBD Shark, *n* = 130; Hash, *n* = 130).

To explore the relevance of cannabinoids’ effect on the microbiome structure of *C. sativa*, we first sought to identify global differences using beta- and alpha-diversity analysis between cultivars (CBD Yummy, CBD Shark and Hash), irrespectively of growth stage and plant part. Qualitative beta-diversity was calculated with Bray–Curtis dissimilarity to identify differential clustering in principal coordinate analysis (PCoA) between groups. Alpha diversity was calculated using Shannon’s index which takes into account both the richness and evenness of groups. Taken together, Bray–Curtis beta-diversity of the fungal (ITS) microbiome showed statistically significant dissimilarity between cultivars (PERMANOVA: *R*^2^ = 0.013, *P* = 0.001) albeit no differences in richness and evenness was measured by the Shannon alpha-diversity metric (Figure 1A and Supplementary Figure S2C). Although statistically significant, the clustering of cultivar level differences on the PCoA plot was seemingly ambiguous (PERMANOVA: *R*^2^ = 0.013, *P* = 0.001), hinting toward more subtle punctual and spatial alterations in the microbiome (Figure 1A). Cannabinoid synthesis is gradually increased during the life cycle of the plant with peak concentrations on the inflorescence and sweet leaves during the late flowering stage (Aizpurua-Olaizola et al., 2016; Small, 2017). Correlatively, clear spatio-temporal variations in beta- and alpha-diversity metrics were observed for the fungal microbiome (Figures 1B–E). There was clear growth stage level clustering in the fungal microbiome (PERMANOVA: *R*^2^ = 0.080, *p* = 0.001), and slightly more intimate grouping between the early and late flowering stages (PERMANOVA: pseudo-*F* = 4.89) as opposed to the pre-vegetative (PERMANOVA: pseudo-*F* = 11.70) and propagation stages (PERMANOVA: pseudo-*F* = 10.32) (Figure 1B). Additionally, the propagation clustering was further removed from the other growth stages, including the pre-vegetative stage (PERMANOVA: pseudo-*F* = 16.29). As the cloned plants are initially grown in rock wool during the propagation stage (rather than coco), this clustering away from the coco substrate grown plants was expected (Figure 1B). The clustering of plant parts was also apparent and distinct (PERMANOVA: *R*^2^ = 0.091, *p* = 0.001), with greater dissimilarity between the belowground microbiome (Rhizosphere, endorhizosphere) and the aboveground microbiome (leaves, sweet leaves, and inflorescence), most notably between the rhizosphere and leaves (PERMANOVA: pseudo-*F* = 21.13) (Figure 1C). The Shannon alpha-diversity of fungal populations progressively increased as the plant aged and appeared to level off at the pre-vegetative stage and slightly decreased at the later growth stages (Figure 1D). The fungal microbiome of the rhizosphere presented the highest Shannon index followed by the endorhizosphere and phyllosphere (Figure 1E). No statistically significant differences were observed between the fungal aboveground microbiome (Figure 1E). These results indicate that the microbiome of *C. sativa* increases in richness and evenness as the plant matures and decreases spatially in these features as the connection with the host becomes more intimate and selective. To better characterize the fungal microbiome of each cultivars, we assigned taxonomy to the ASV and calculated their relative frequency (Figure 1F). The phylum Ascomycota and Basidiomycota make up the bulk of the fungal microbiome, with a greater penchant for Ascomycota (Figure 1F). More precisely, of the phylum Ascomycota, the genera *Penicillium*, *Zopfiella*, *Aspergillus*, and *Fusarium* were dominant.

### Diversity Metrics and Taxonomy of the Cannabis Bacterial Microbiome

**FIGURE 2:**
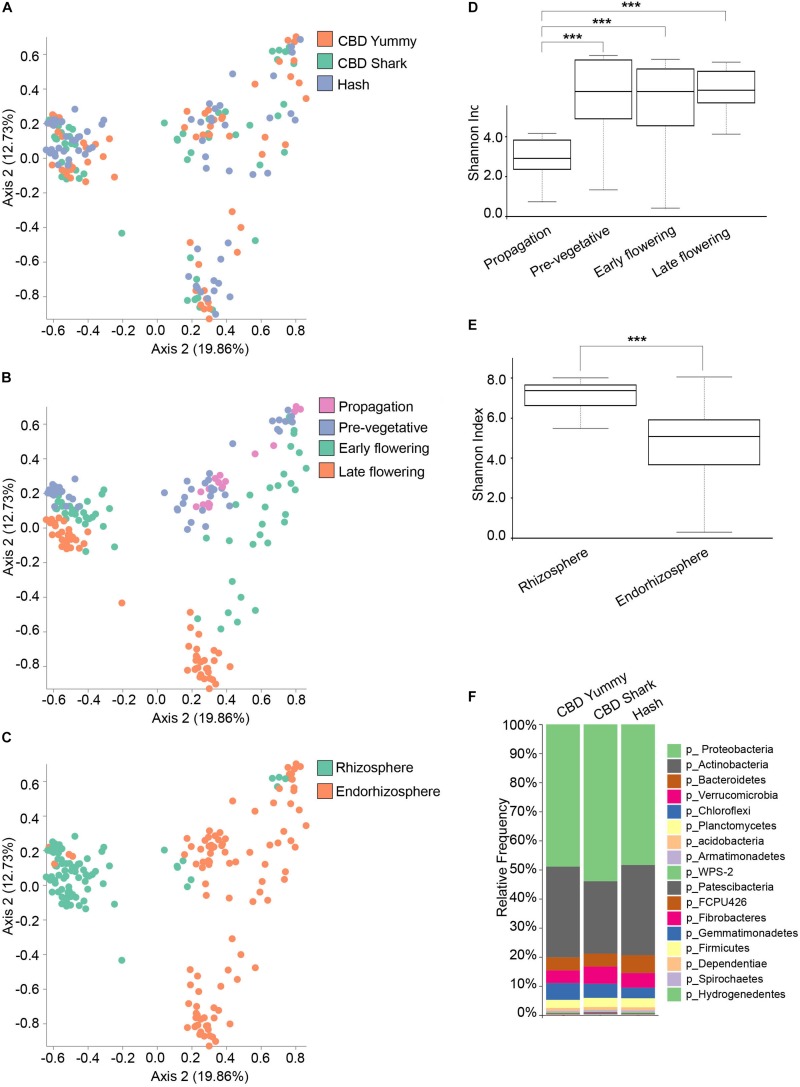
Diversity metrics and taxonomical differences in the bacterial microbiome of cannabis. **(A)** PcoA plot representative of inter-cultivar beta-diversity utilizing Bray–Curtis dissimilarity among samples (PERMANOVA: *R*^2^ = 0.018, *p* = 0.001). Each point represents a sample (CBD Yummy, *n* = 69; CBD Shark, *n* = 69; Hash, *n* = 70). **(B)** PcoA plot representative of temporal beta-diversity utilizing Bray–Curtis dissimilarity among samples (PERMANOVA: *R*^2^ = 0.18, *p* = 0.001). Each point represents a sample (Propagation, *n* = 30; Pre-vegetative, *n* = 60; Early flowering, *n* = 58; Late flowering, *n* = 60). **(C)** PcoA plot representative of spatial beta-diversity utilizing Bray–Curtis dissimilarity among samples (PERMANOVA: *R*^2^ = 0.12, *p* = 0.01). Each point represents a sample (Rhizosphere, *n* = 88; Endorhizosphere, *n* = 120). For all PcoA plots, axis 1 and axis 2 represent the percentage of variance explained by each coordinate. **(D**,**E)** Alpha-diversity metric (Shannon’s index) of temporal and spatial variations amongst groups, respectively. Kruskal–Wallis pair-wise test was used to assess statistical significance between groups (***p < 10e-04,**p < 0.001,*p < 0.05 only statistically significant values are shown, non-significant groups are not represented). **(F)** Bar plot of the relative frequency of bacterial taxa at the phylum level in the three chemotypes studied (CBD Yummy, *n* = 69; CBD Shark, *n* = 69; Hash, *n* = 70).

Conversely, the bacterial (16S) microbiome clustering on PCoA plots only showed significant differences between CBD Shark and Hash (PERMANOVA: *p* = 0.007; PERMANOVA: 0.018, *p* = 0.001) with no differences in the Shannon alpha-diversity metric (Figure 2A and Supplementary Figure S2D), once again hinting toward more subtle punctual and spatial alterations in the microbiome (Figure 2A). As with the fungal microbiome, the bacterial microbiome also presented the same overt spatio-temporal clustering (PERMANOVA, temporal: *R*^2^ = 0.18, *p* = 0.001; PERMANOVA, spatial: *R*^2^ = 0.12, *p* = 0.01) (Figures 2B,C). The Shannon index of the bacterial community plateaued at the pre-vegetative stage and no differences in richness and evenness could be observed between this stage and the later stages (Figure 2D). Although we were unable to extract bacterial DNA from the phyllosphere, a similar trend (significant differences in diversity) was observed when comparing the bacterial and the fungal microbiomes associated with the rhizosphere and the endorhizosphere (Figure 2E). Interestingly, as no 16S could be amplified from the phyllosphere at all growth stages, we hypothesize that the indoor facility or possible interaction between the fungal and bacterial microbiome in this environment might have modulated (possibly attenuated) the rise of a bacterial phyllosphere microbiome; however, this exceeds the intent of the present work and will not be explored further. To better characterize the bacterial microbiome of each cultivar, we assigned taxonomy to the ASV and calculated their relative frequency, which highlighted Proteobacteria and Actinobacteria as the dominant phyla (Figure 2F).

### Intra- and Inter-Cultivar Variations in the Cannabis Fungal Microbiome

**FIGURE 3:**
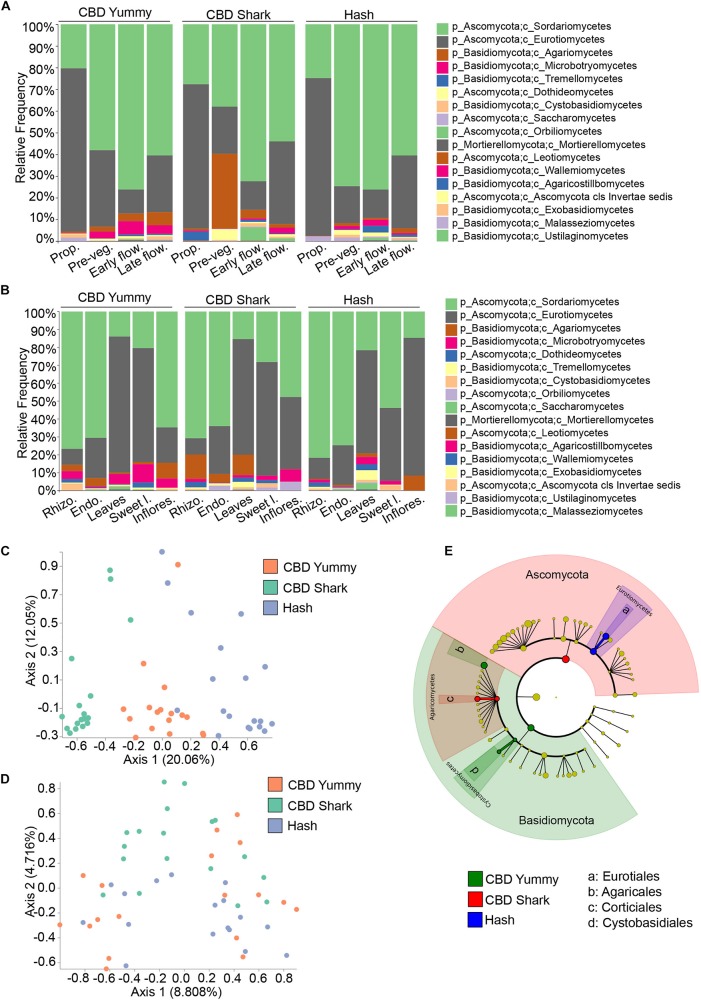
Intra- and inter-cultivar differences in the fungal microbiome of cannabis along spatio-temporal lines. **(A)** Bar plot of the relative frequency of fungal taxa at the phylum and class level in the three chemotypes studied (CBD Yummy, *n* = 130; CBD Shark, *n* = 130; Hash, *n* = 130) at different growth stages (Prop = Propagation; Pre-veg = Pre-vegetative; Early flow = Early flowering; Late flow = Late flowering) and **(B)** for different plant parts (Rhizo = Rhizosphere; Endo = Endorhizosphere; Sweet l = Sweet leaves; Inflores = Inflorescence). Statistically significant differences in taxa between groups were measured using ANCOM. **(C)** PcoA plot representative of inter-cultivar beta-diversity utilizing Bray–Curtis dissimilarity (PERMANOVA: *R*^2^ = 0.22, *p* = 0.001) and **(D)** Jaccard similarity among samples (PERMANOVA: *R*^2^ = 0.058, *p* = 0.001). Each point represents a sample (CBD Yummy, *n* = 70; CBD Shark, *n* = 70; Hash, *n* = 70). **(E)** Taxonomic cladogram obtained from LEfSe analysis showing belowground fungal taxa that are differentially abundant between cultivars at the late flowering stage. Green represents an increased abundance in CBD Yummy, red an increased abundance in CBD Shark and blue an increased abundance in Hash chemotype.

To further investigate spatio-temporal taxonomic differences in the fungal microbiome of *C. sativa* we first looked into intra-cultivar and inter-cultivar variations (Figures 3A,B). After assigning taxonomy to groups, we applied Analysis of composition of microbiome (ANCOM) to highlight significantly different taxonomic classes (Mandal et al., 2015). Across cultivars, the relative frequency of the fungal class Sordariomycetes steadily increased from the propagation phase to the early flowering phase and then slightly decreased during the late flowering stage, while the class Eurotiomycetes followed an opposing trend (Figure 3A). Apart from this pattern, the fungal taxonomic profile of each cultivar matured differently in time (Figure 3A). Interestingly, already at the propagation phase, the taxonomic profiles of each cultivars were different – possibly hinting to a form of microbiome transmission from the mother plants as a result of the cloning process. Notably, the class Tremellomycetes had a significantly higher frequency in CBD Shark at the propagation stage accompanied by a unique and dramatic switch to the class Agaromycetes at the pre-vegetative stage (Figure 3A). Because the cultivars do not readily produce cannabinoids at these growth stages, relative differences in taxonomy cannot be attributed solely to their biosynthesis but may be the result of another unknown genotype-dependent selective pressure. At high cannabinoid producing growth stages, inter-cultivar differences were observed between the low THC chemotype (CBD Yummy) and the mid to high THC chemotypes (CBD Shark and Hash, respectively) (Figure 3A). At the early flowering stage, Sporidiobolales of the class Microbotryomycetes and Corticiales of the class Agaricomycetes had a higher relative frequency in CBD Yummy when compared to mid- to high-THC chemotypes (CBD Shark and Hash, respectively). At the late flowering stage Agaricales of Agaricomycetes also had a higher frequency in CBD Yummy relative to mid- to high-THC chemotypes (Figure 3A).

The different microenvironments of all the cultivars, as previously mentioned, were dominated by Ascomycota, more precisely of the orders Sordariomycetes and Eurotiomycetes. Intriguingly, the relative frequency of these fungi was similar in the rhizosphere, endorhizosphere, and inflorescence of the phyllosphere, which are seemingly disparate microenvironments. The leaves and sweet leaves of the phyllosphere had the lowest relative frequency of Sordariomycetes and a higher relative frequency of Eurotiomycetes (Figure 3B). Comparing each microenvironment between cultivars using ANCOM, we observed changes in Basidiomycota, more specifically of the class Agaricomycetes. More precisely, of the class Agaricomycetes, the rhizosphere and endorhizosphere presented cultivar level differences in the order Corticiales whilst the leaves presented cultivar level differences in Polyporales and the inflorescence in Agaricales (Figure 3B). Belowground, Corticiales was more abundant in CBD Shark and gradually less abundant in CBD Yummy followed by Hash. Polyporales had a higher relative frequency on the leaves of CBD Shark and very low relative frequency in the other cultivars. Agaricales was abundant on the inflorescence of CBD Yummy and progressively diminished in abundance in CBD Shark and Hash (Figure 3B).

Although differences in fungal taxa were observed between cultivars on all plant parts in a temporal fashion, to our surprise, the highest diversity was seen belowground (rhizosphere and endorhizosphere) where no appreciable amounts of cannabinoids have been detected (Figures 3C,D). Restricting our analysis of beta-diversity to specific plant parts at specific time points, we were surprised to discover that significant diversity was seen solely belowground at the pre-vegetative and flowering stages. Restricting our analysis of Bray–Curtis dissimilarity to the belowground microbiome at the pre-vegetative stage, we observed a clear and distinct clustering driven by the cultivars (PERMANOVA: *R*^2^ = 0.22, *p* = 0.001) (Figure 3C). This gave a better perception of cultivar level differences when compared to the more generalized view confounding growth stage and plant part (PERMANOVA: *R*^2^ = 0.013, *P* = 0.001) (Figure 1A). The use of a quantitative Jaccard beta-diversity metric, which only takes into account the presence or absence of fungi rather than abundance, also shed light on cultivar level clustering of the belowground microbiome at the late flowering stage (PERMANOVA: *R*^2^ = 0.058, *P* = 0.001) (Figure 3D). Using a quantitative measure rather than a qualitative measure gave better insight into strong genotype-dependent variations as it only takes into account the presence or absence of a particular taxa as opposed to a gradient of abundance (Lozupone et al., 2007). The mid THC cultivar (CBD Shark) stands apart from CBD Yummy and Hash in terms of dissimilarity (PERMANOVA: *p* = 0.01 and *p* = 0.001, respectively) (Figure 3D). This observation led us to try and identify the differentially present fungi at the late flowering stage using the biomarker discovery tool applying a linear discriminant analysis effect size (LEfSe) (Segata et al., 2011). LEfSe identified Eurotiales, Agaricales, Corticiales, and Cystobasidiales as cultivar level biomarkers of the belowground microbiome at the late flowering stage, which in part corroborated our ANCOM findings and identified novel differentially abundant taxa (Figure 3E). Eurotiales of the class Eurotiomycetes is prevalent in Hash while Corticiales of the class Agaricomycetes is prevalent in CBD Shark, and Agaricales of the class Agaricomycetes and Cystobasidiales of the class Cystobasidiomycetes are predominant in CBD Yummy (Figure 3E).

### Intra- and Inter-Cultivar Variations in the Cannabis Bacterial Microbiome

**FIGURE 4:**
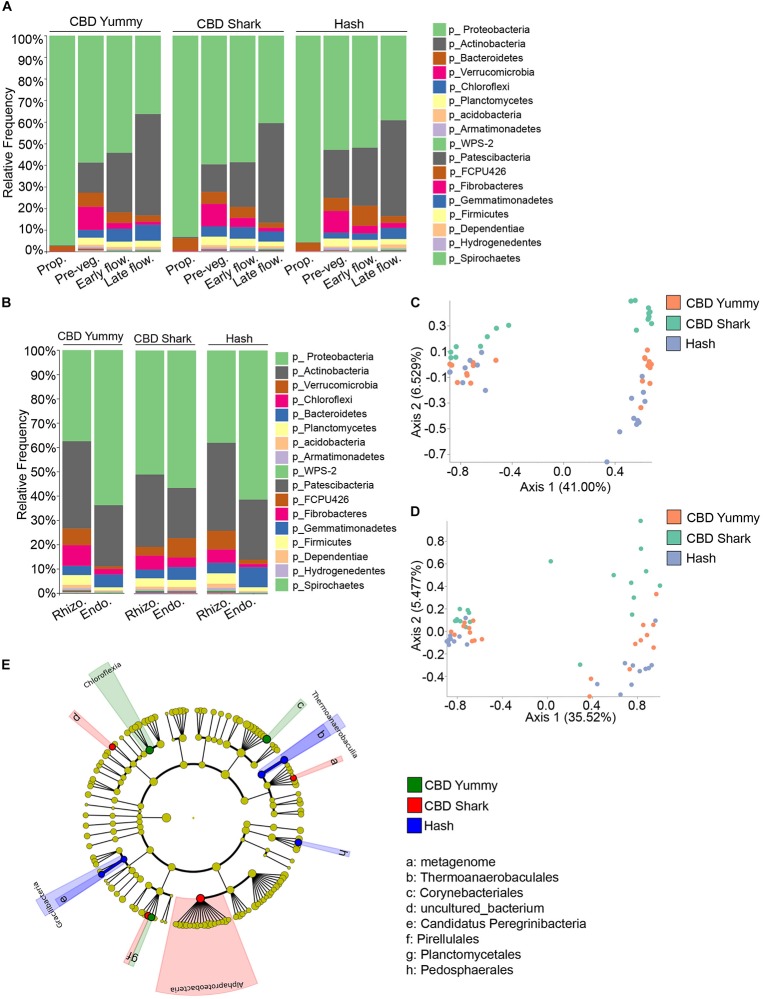
Intra- and inter-cultivar differences in the bacterial microbiome of cannabis along spatio-temporal lines. **(A)** Bar plot of the relative frequency of bacterial taxa at the phylum level in the three chemotypes studied CBD Yummy, *n* = 69; CBD Shark, *n* = 69; Hash, *n* = 70) at different growth stages (Prop = Propagation; Pre-veg = Pre-vegetative; Early flow = Early flowering; Late flow = Late flowering) and **(B)** for different plant parts (Rhizo = Rhizosphere; Endo = Endorhizosphere). Statistically significant differences in taxa between groups were measured using ANCOM. **(C)** PcoA plot representative of inter-cultivar beta-diversity utilizing Bray–Curtis dissimilarity (PERMANOVA: *R*^2^ = 0.073, *p* = 0.039) and **(D)** Jaccard similarity among samples (PERMANOVA: *R*^2^ = 0.067, *p* = 0.021). Each point represents a sample (CBD Yummy, *n* = 70; CBD Shark, *n* = 70; Hash, *n* = 70). **(E)** Taxonomic cladogram obtained from LEfSe analysis showing belowground bacterial taxa that are differentially abundant between cultivars at the late flowering stage. Green represents an increased abundance in CBD Yummy, red an increased abundance in CBD Shark and blue an increased abundance in Hash chemotype.

In the same fashion, inter-cultivar and intra-cultivar spatio-temporal variations were also investigated in the bacterial microbiome. In all cases, the relative frequency of the most abundant phyla, Proteobacteria and Actinobacteria, varied in time. More specifically, Burkholderiaceae and Rhizobiaceae (of the Proteobacteria phylum), and Streptomycetaceae and Norcardioidaceae (of the Actinobacteria phylum) dominated the plant microbiome. Proteobacteria lessened gradually from the propagation phase to the late flowering stage while Actinobacteria progressively increased (Figure 4A). Unlike the fungal microbiome, the temporal taxonomic profiles between cultivars was quite similar, with comparable microbiomes at the propagation stage consisting primarily of Proteobacteria and Bacteroidetes (Figure 4A). In all cases, the relative frequency of the phylum Bacteroidetes and Verrucomicrobia increased from the propagation to the pre-vegetative stage to then slightly drop during subsequent growth stages. Furthermore, the relative frequency of Chloroflexi was maintained or slightly augmented from the pre-vegetative stage to later stages (Figure 4A). While the global bacterial microbiome seemed to diminish in relative frequency to be taken over by the phylum Actinobacteria, taxa with a lower overall abundance, such as WPS-2 and Patescibacteria, increased in abundance from the pre-vegetative to the later stages (Figure 4A). As these temporal variations were limited to the belowground microbiome (rhizosphere and endorhizosphere), all inter-cultivar differences are seemingly independent of cannabinoid signaling.

As we were unable to extract appreciable bacterial DNA from the phyllosphere, our analysis of bacterial diversity and taxonomic classification of spatial differences was limited to the rhizosphere and endorhizosphere. The taxa identified in the rhizosphere and endorhizosphere were similar to one another and were comprised of the same phyla but with varying relative frequencies; with the exception of CBD Shark which seemed to be an outlier to the tendency emulated by CBD Yummy and Hash (Figure 4B). In all cultivars, the relative frequency of the phylum Proteobacteria was greater in the endorhizosphere while the phylum Actinobacteria followed the opposite trend but with only a diminutive decline. Furthermore, CBD Shark saw an augmentation of the phylum Chloroflexi in the endorhizosphere while the other cultivars saw a significant drop. This same tendency was also observed for the phylum FCPU426, Armatimonadetes and Acidobacteria (Figure 4B). Cannabinoids being largely exempt from the belowground microbiome, fluctuations observed in the bacterial microbiome cannot be attributed solely to their production and are at least partially independent.

Belowground bacterial microbiome dissimilarity between cultivars was measured using beta-diversity at the pre-vegetative and late flowering stage (Figures 4C,D). Differential clustering between cultivars at the pre-vegetative stage was demonstrated by applying the Bray–Curtis beta-diversity metric, while differential clustering at the late flowering stage was demonstrated by the Jaccard beta-diversity metric (PERMANOVA: *R*^2^ = 0.073, *p* = 0.039 and *R*^2^ = 0.067, *p* = 0.021, respectively) (Figures 4C,D, respectively). In both cases, only CBD Shark and Hash represented statistically significant dissimilarity driven by the cultivars (PERMANOVA: *p* = 0.029 and *p* = 0.035, respectively) (Figures 4C,D). We then utilized LEfSe analysis to pinpoint cultivar level differences in the bacterial microbiome (Segata et al., 2011). LEfSe identified 8 differentially abundant taxa including bacteria from the class Chloroflexia, Thermoanaerobaculia, Gracilibacteria, and Alphaproteobacteria (Figure 4E).

### Spatio-Temporal Variations in Predicted Pathways of the Cannabis Bacterial Microbiome

**FIGURE 5:**
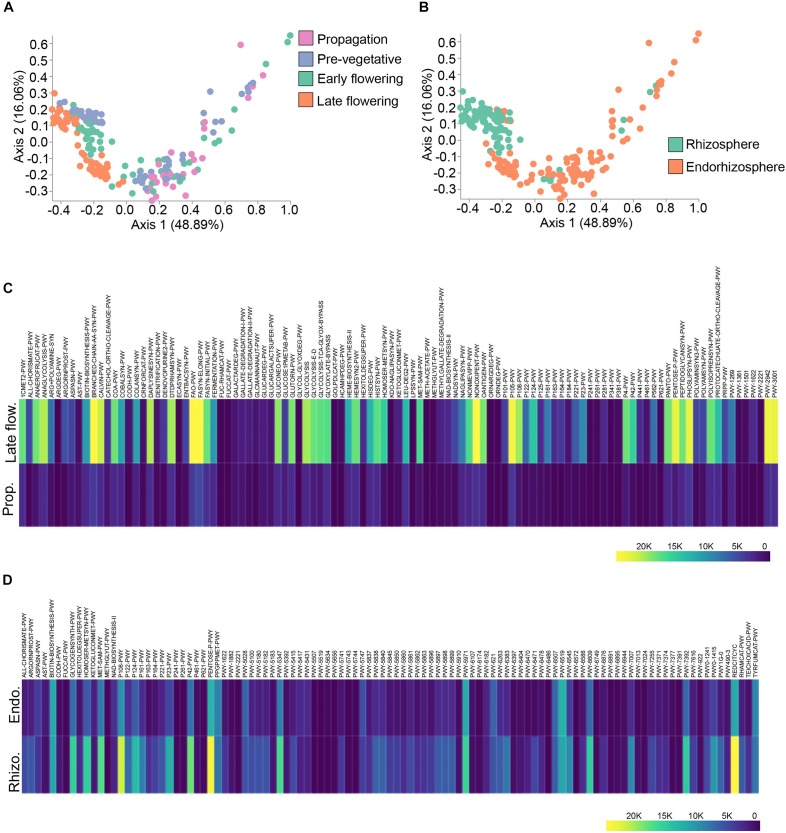
Spatio-temporal variations in PICRUSt predicted pathways. **(A)** PcoA plot representative of temporal beta-diversity utilizing Bray–Curtis dissimilarity metric among samples (PERMANOVA: *R*^2^ = 0.24, *p* = 0.001). Each point represents a sample (Propagation, *n* = 30; Pre-vegetative, *n* = 60; Early flowering, *n* = 58; Late flowering, *n* = 60). **(B)** PcoA plot representative of spatial beta-diversity utilizing Bray–Curtis dissimilarity among samples (PERMANOVA: *R*^2^ = 0.20, *p* = 0.001). Each point represents a sample (Rhizosphere, *n* = 88; Endorhizosphere, *n* = 120). For all PcoA plots, axis 1 and axis 2 represent the percentage of variance explained by each coordinate. **(C**,**D)** Heatmap of differentially abundant pathways predicted by PICRUSt along temporal and spatial lines, respectively. Statistically significant differences between groups were measured using ANCOM. Prop = Propagation; Late flow = Late flowering; Rhizo = Rhizosphere; Endo = Endorhizosphere.

To better link the spatio-temporal differences in bacterial taxa to predicted functions, we used the analytic pipeline Phylogenetic Investigation of Communities by Reconstruction of Unobserved States (PICRUSt) (Langille et al., 2013). The output from PICRUSt gave relative abundance of MetaCyc pathways which can be further processed in the QIIME2 pipeline. The predicted pathways were hence run through Bray–Curtis dissimilarity to visualize clustering along the spatio-temporal axis (Figures 5A,B). Predicted functions clustered at discrete growth stages (PERMANOVA: *R*^2^ = 0.24, *p* = 0.001) with the most differential clustering being between the propagation and late flowering stages (PERMANOVA: pseudo-*F* = 64.88, *p* = 0.001) (Figure 5A). Predicted functions also clustered dependent on microenvironment (PERMANOVA: *R*^2^ = 0.20, *p* = 0.001), that is to say between the rhizosphere and endorhizosphere (PERMANOVA: pseudo-F = 49.95, *p* = 0.001) (Figure 5B). ANCOM was employed to delineate differentially abundant predicted pathways between the propagation stage and late flowering stage as well as spatial differences between the rhizosphere and endorhizosphere. The top one hundred differentially abundant pathways were incorporated into a heatmap using Plotly (Figures 5C,D). The late flowering stage strikingly had a greater abundance in multiple pathways while the propagation stage was minimalist in comparison. This led us to conclude that as the plant ages, its microbiome and its predicted collective metagenomic functions are intimately linked and are altered in accordance (Figure 5C). We further attempted to identify the bacterial functions that may help them thrive in the rhizosphere and endorhizosphere of *C. sativa*. In the endorhizosphere, as opposed to the rhizosphere, predicted functions seemed to globally diminish in relative abundances (Figure 5D). The most abundant predicted pathways that varied spatio-temporally were of lipid metabolism which included PHOSLIPSYN-PWY (super-pathway of phospholipid biosynthesis I), FASYN-ELONG-PWY (fatty acid elongation) and FAO-PWY (fatty acid beta-oxidation); amino acid metabolism, including PWY-3001 (super-pathway of L-isoleucine biosynthesis), PWY-2942 (L-lysine biosynthesis III), and BRANCHED-CHAIN-AA-SYN-WY (super-pathway of branched chain amino acid biosynthesis); and glucose and pentose metabolism, including PENTOSE-P-PWY (pentose phosphate pathway), NON-OXIPENT-PWY (Pentose phosphate pathway non-oxidative branch), P105-PWY (TCA cycle IV) and REDCITCYC (TCA cycle VI) (Figures 5C,D). Overall, a great deal of glycolysis pathways were amongst the most abundant pathways (Figure 5C). Although these pathways may give insight into metabolic functions of the *C. sativa* microbiome, the implication of these pathways remains to be explored as they are only marker-based predictions and are not validated functions.

## Discussion

The present study successfully identified spatio-temporal and cultivar level variations in the fungal and bacterial microbiomes of *C. sativa.* The beta-diversity metrics demonstrated clear dissimilarly between the microbiome at different growth stages and across micro-environments. Alpha-diversity demonstrated a concomitant rise in community richness and evenness as the plant ages, but a drop in those features was progressively observed between the rhizosphere, endosphere and phyllosphere. The latter is in accord with previous work showing a selection model where each compartment acquires a subset from the others (Edwards et al., 2015). Inter-cultivar diversity was also discovered. To our surprise, the greatest diversity between chemotypes was found belowground where we were able to identify cultivar specific inhabitant using LEfSe. Although the aim of the study was to correlate chemotype-dependent cannabinoid production to alterations in the microbiome, it would seem that cannabinoid independent mechanisms may, at least in part, modulate the structure and function of the microbiome. In fact, the inter-cultivar variations are most striking belowground at the pre-vegetative stage where no appreciable amounts of cannabinoids are produced. Analogously, inter-cultivar differences in the fungal microbiome are evident at the non-cannabinoid producing stages (propagation phase and pre-vegetative stage). This observation also led us to postulate a seed-independent vertical mode of microbiome transmission via the cloned mother plant (Hardoim et al., 2012; Berg and Raaijmakers, 2018). These early settlers are known for having a lasting impact on the evolving microbiome (a priority effect) through niche pre-emption and niche modification and thus should warrant further investigation (Santoyo et al., 2016; Niu et al., 2017; Berendsen et al., 2018). Of note, the CBD Shark microbiome has been a frequent outlier in terms of microbiome maturation and diversity when compared to the other cultivars. The uniqueness of this chemotype also merits further investigation.

Bacterial communities within the plant microbiome adapt their metabolism to their changing physicochemical environment (Chaparro et al., 2013; Zhalnina et al., 2018). Using PICRUSt, we identified spatio-temporal variations in the predicted abundance of bacterial pathways. Interestingly, predicted pathways related to the metabolism of carbohydrates were dominant, a feature that is known of plant-associated microbes (Levy et al., 2017). However, these remain marker-based predictions and are not validated functions.

Apart from the production of cannabinoids, *C. sativa* is renowned for the production of an assortment of secondary metabolites that may be dominant modulators of the microbiome (Kim and Mahlberg, 1997; Elsohly and Slade, 2005; Flores-Sanchez et al., 2012; Staginnus et al., 2014; Aizpurua-Olaizola et al., 2016). In fact, CBD Yummy, CBD Shark and Hash not only vary in their production of cannabinoids but also in their production of unique terpenes, namely pinene, linalool, limonene, and humulene. Furthermore, as most are looking at the trichome rich above ground leafy growth, there is an unexplored richness hidden in rhizosphere and endorhizosphere of the plant. Future studies aiming to identify the chemotype dependent secondary metabolites produced and exuded in these microenvironments, as well as their genetic disposition, could better explain these variations and also possibly identify novel plant genes and pathways responsible for the modulation of the microbiome structure and function. More probably, inter-cultivar variations in the microbiome are the result of multiple factors that dynamically interact with one another.

Although the genotype has strong selective pressure over the microbiome, the soil substrate remains the primary factor dictating the composition of the plant microbiome (Bulgarelli et al., 2012; Peiffer et al., 2013; Edwards et al., 2015; Qiao et al., 2017). Hence, variations in the identified microbes of *C. sativa* between studies is to be expected. Notably, apart from the high prevalence of Ascomycota, the majority of bacterial and fungal taxa identified by other studies are of very low abundance or absent in the present work (Kusari et al., 2012; Winston et al., 2014; Scott et al., 2018). Our experiment being conducted in an indoor commercial setting, the plants we sampled did not have much to contend with in terms of major biotic and abiotic stressors when compared to plants grown outside. As plants differentially recruit their microbiome under stress, *C. sativa* grown outdoors may also drastically change its microbiome in accordance (Rasmann et al., 2005; Dicke, 2009). Aside from the edaphic and environmental factors, the use of DADA2 for quality control which outputs AVS rather than operational taxonomic units (OTUs) might be of some relevance (Callahan et al., 2016). Unfortunately, we were unable to extract any significant bacterial DNA from the phyllosphere for comparison’s sake. As the study remains descriptive in nature, future work should aim at describing mechanistic and molecular interactions between the host and its microbiome to find causation. Such discoveries could help in engineering a plant growth and health promoting microbiome in economically important crops.

## Data Availability Statement

All sequences generated in this study have been deposited in DDBJ/EMBL/GenBank under the BioSample Accessions: PRJNA595913.

## Author Contributions

DC responsible for part of the sampling, all metagenomic analysis, and writing of the manuscript. AN responsible for part of the sampling and editing of the manuscript. MF and DJ supervised and conceived the project, and edited the manuscript.

## Conflict of Interest

The authors declare that the research was conducted in the absence of any commercial or financial relationships that could be construed as a potential conflict of interest.
