# Supplementary figures and images for: Spatio-Temporal and Cultivar-Dependent Variations in the Cannabis Microbiome
Source: Front Microbiol. 2020 Mar 24;11:491. doi: 10.3389/fmicb.2020.00491 (PMC7105690; doi:10.3389/fmicb.2020.00491)

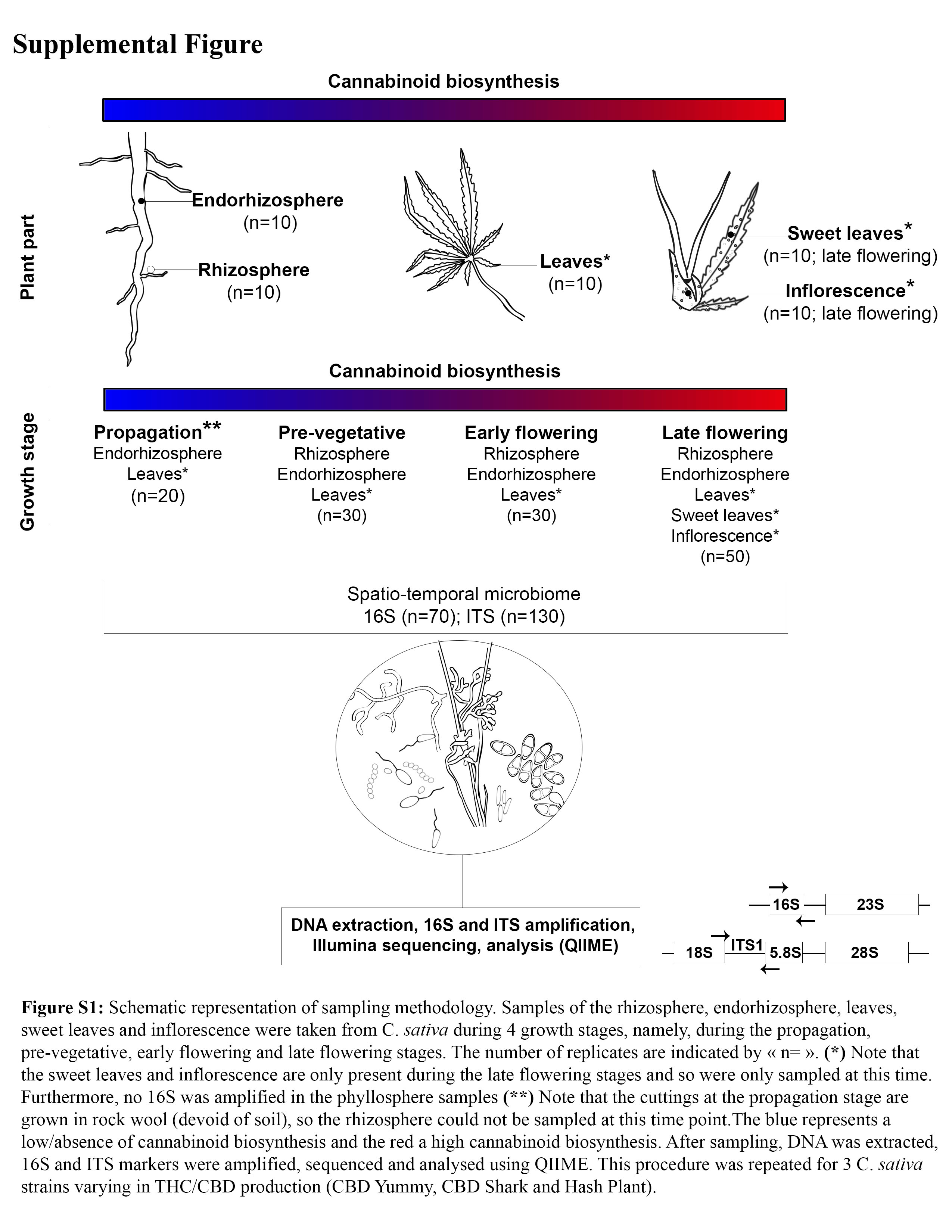

Supplement: Supplementary file 1 [file Image_1.JPEG]

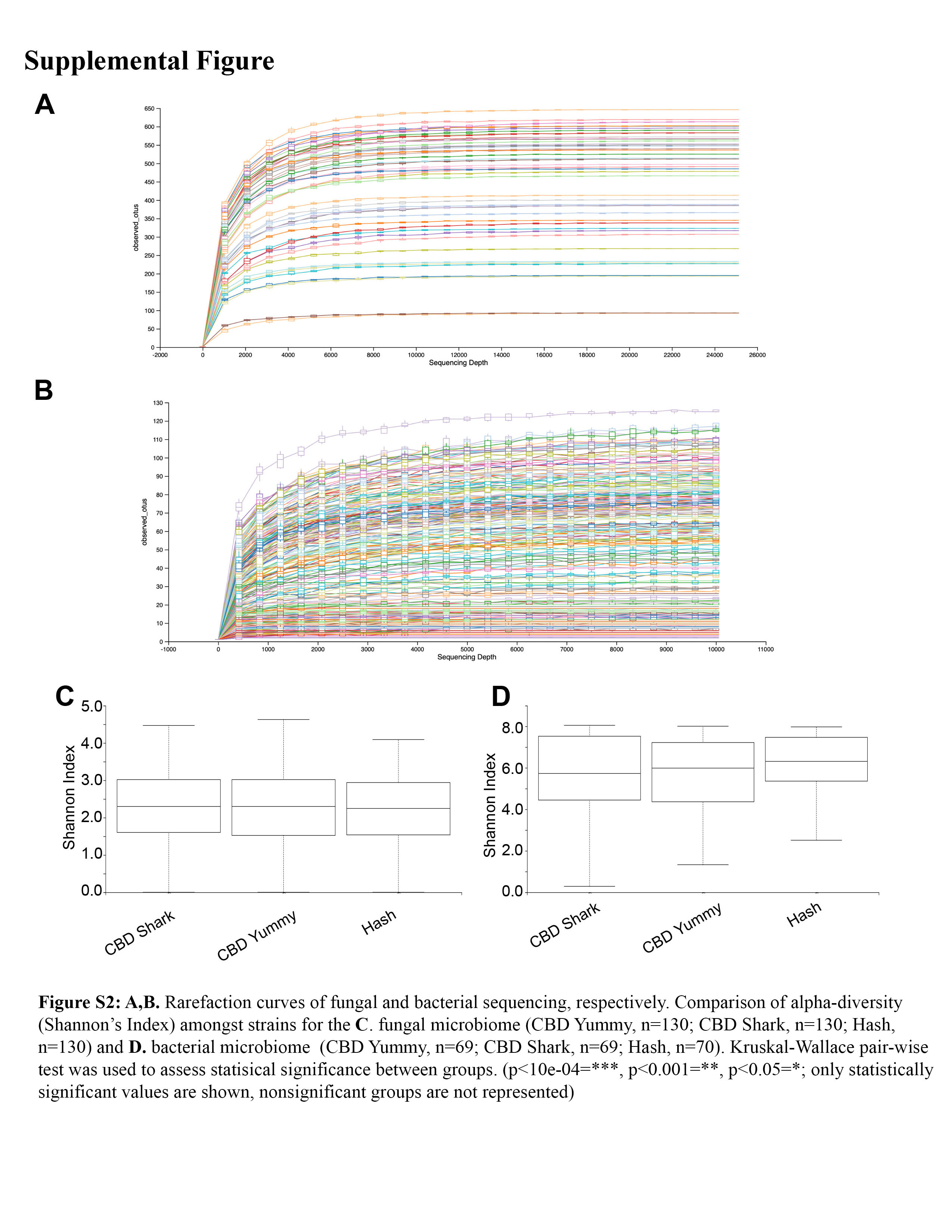

Supplement: Supplementary file 2 [file Image_2.JPEG]
